# Incidence rates of immune-related adverse events and their correlation with response in advanced solid tumours treated with NIVO or NIVO+IPI: a systematic review and meta-analysis

**DOI:** 10.1186/s40425-019-0779-6

**Published:** 2019-12-04

**Authors:** Puyuan Xing, Fan Zhang, Guoqiang Wang, Yu Xu, Chengcheng Li, Shouzheng Wang, Yiying Guo, Shangli Cai, Yan Wang, Junling Li

**Affiliations:** 1 Department of Medical Oncology, National Cancer Center/National Clinical Research Center for Cancer/Cancer Hospital, Chinese Academy of Medical Sciences and Peking Union Medical College, 17 Pan-jia-yuan South Lane, Chaoyang District, Beijing, 100021 China; 2 The Department of Oncology, Chinse PLA General Hospital, Beijing, China; 3 The Medical Department, 3D Medicines Inc, Shanghai, People’s Republic of China

**Keywords:** Immune-related adverse events, Meta-analysis, Nivolumab, Ipilimumab

## Abstract

**Background:**

Deciphering the correlation between immune-related adverse events (irAEs) categorized by organ system class and clinical benefit of immunotherapy is critical for clinical practice. The aim of this study is to investigate the incidence rates of irAEs and their correlations with objective response rate (ORR) in patients with advanced solid tumours treated with nivolumab (NIVO) or nivolumab plus ipilimumab (NIVO+IPI).

**Methods:**

PubMed, Embase and Cochrane library were searched for eligible studies from January 1st, 2000 to May 1st 2019. Published clinical trials on NIVO or NIVO+IPI with reported irAEs were included. Logit transformation of the irAE incidences was applied for the generation of pooled estimate and Pearson correlation coefficient was calculated to evaluate the correlation between irAE and ORR.

**Results:**

48 clinical trials involving 7936 patients treated with NIVO or NIVO+IPI were included. Compared to NIVO, NIVO+IPI led to more all-grade and grade 3 or higher irAEs categorized by system organ class (*P* < 0.05). The ORR of NIVO was positively correlated with the incidence rate of skin (r = 0.79, *P* < 0.001), gastrointestinal (r = 0.56, *P* = 0.006) and endocrine irAEs (r = 0.44, *P* = 0.05), but not hepatic, pulmonary and renal irAEs. The ORR of NIVO+IPI was positively correlated with the incidence rate of skin (r = 0.54, *P* = 0.04), and gastrointestinal irAEs (r = 0.60, *P* = 0.02), but not endocrine, hepatic, pulmonary and renal irAEs.

**Conclusion:**

This meta-analysis summarizes the incidence rates of irAEs in patients with advanced solid tumours treated with NIVO or NIVO+IPI, and uncovers their correlations with ORR across multiple neoplasms. These findings highlight the potential of irAE to reflect response to NIVO or NIVO+IPI.

## Introduction

For the past decades, immunotherapy by targeting programmed cell death-1 (PD-1), programmed cell death-ligand 1 (PD-L1), or cytotoxic T lymphocyte associated antigen 4 (CTLA-4) has revolutionized the treatment of cancer. Among these regimens, nivolumab monotherapy (NIVO) or nivolumab plus ipilimumab (NIVO+IPI) have been approved by the US Food and Drug Administration for indications including advanced lung cancer, melanoma, renal cell carcinoma (RCC), head and neck squamous cell carcinoma (HNSCC), hepatocellular carcinoma (HCC), urothelial carcinoma, colorectal carcinoma (CRC) and classical Hodgkin lymphoma. Despite the impressive anti-tumour activity by removing the barrier of immune checkpoint, anti-PD-1/PD-L1 and anti-CTLA-4 reactivate the T cell-mediated anti-tumour immunity, and meanwhile, inevitably break the innate immuno-homeostasis via facilitating the loss of immune tolerance to autoantigens [1], which is associated with the generation of adverse events, known as immune-related adverse events (irAEs).

IrAEs are varied in terms of tissues affected, the severity, and the time of onset relative to the initiation of treatment [2–8]. Numerous clinical trials have outlined a crude profile of irAEs, including skin, gastrointestinal, pulmonary, hepatic, endocrine and renal toxicities [1, 9]. The most common irAEs include pruritus, rash, nausea, diarrhea and thyroid disorders [9]. The vast majority of these irAEs develop within the first few weeks to months after treatment initiation, while others like liver toxicity or hypophysitis appear later [1, 10]. Most irAEs are mild to moderate, except some are potentially fatal, e.g., colitis, pneumonitis, hepatitis, myocarditis, and neurotoxic effects [11]. Hence, there is an urgent need to be acquainted with the toxicological profile. A recent meta-analysis including 125 clinical trials, provides a comprehensive profile for the irAEs of single-agent immunotherapy [12]. However, this meta-analysis merely provided the pooled estimate of irAEs from all anti-PD-1/PD-L1 monotherapy, without announcing the specific data for each one and comparing the difference among these agents. In addition, severer irAEs owing to the combination with anti-CTLA-4 worth more awareness [12], and thus the incidence rates of irAEs for NIVO and NIVO+IPI are of great vitality and remain to be studied.

Not only do irAEs belong to side effects that require intensive care, they also serve as windows into the anti-tumour response of ICIs. The association between irAEs and survival of patients treated with NIVO was first reported in melanoma. In 148 patients with melanoma treated with NIVO, irAEs were associated with better OS using a 12-week landmark [13]. Recent studies have also demonstrated that in non-small cell lung cancer (NSCLC) treated with NIVO, the patients with irAE occurring within 6 weeks post treatment, reached longer progressive-free survival (PFS) and overall survival (OS) than those without irAE [14]. However, irAEs of different organs vary in severity and time of onset [2–8]. The irAEs of grade 3 or 4 according to CATAE v4.0 demand extra usage of prednisone and reduction of ICI dose (or even cessation) [15, 16], relative to poorer prognosis of ICIs [12, 15–18]. Several irAEs are late-onset [3–8], appearing after the confirmation of objective response, which might result in lower relativity. Whether the irAEs of different organs contribute equally in the association with ICI benefit needs to be explored. Besides, whether the association between irAEs and survival benefit can be applied to other tumour types needs to be further studied.

In order to address these issues above, we did this meta-analysis to depict the landscape of irAE incidence rates and to investigate their correlations with the response in patients with advanced solid tumours treated with NIVO or NIVO+IPI.

## Methods

### Search strategy and study selection

The present review was prepared according to Preferred Reporting Items for Systematic reviews and Meta-Analyses (PRISMA) [19]. A systematic search of the literature was conducted to identify published clinical trials of nivolumab with or without ipilimumab that reported treatment-related irAEs and objective response rate (ORR). The search was performed using Embase, PubMed, and Central Register of Controlled Trials of the Cochrane Library. The following free language terms and medical subject headings (MeSH) were used as the specific search strategy: “nivolumab”, “ipilimumab” and “clinical trial”. The last search was updated on May 1st, 2019. Studies eligible for inclusion met all of the following criteria: (1) clinical trials of solid tumours (2) participants were treated with single-agent NIVO or NIVO+IPI, (3) reported tabulated data on treatment-related irAEs, and (4) published in English. The studies published online ahead of print were eligible, but meeting abstracts were excluded. When multiple publications reporting on the same study population were identified, the one with most updated and/or comprehensive adverse event data was selected.

### Data extraction

The information of the first author’s names, the year of publication, the name of journal and trial, cancer type, the phase of trial, dosing schedule, the number of patients, the percentage of patients according to age and sex, the number of irAEs, the criteria for adverse events reported in the publication, ORR and the criteria for ORR evaluation were extracted from each included study. The data of both all-grade (severity) and grade 3 or higher irAEs were extracted. Commonly select treatment-related AEs of a possible immunologic etiology were categorized by system organ class (according to common terminology criteria adverse events, version 4.0 [CATAE v4.0]), including skin irAEs (rash, pruritus, vitiligo, dry skin, dermatitis acneiform, erythema, rash maculopapular, dermatitis, rash acneiform, skin hypopigmentation, eczema, rash popular, generalized rash, urticarial, and palmar-plantar erythrodysasthesia syndrome), endocrine irAEs (hypothyroidism, hyperthyroidism, hyperglycemia, blood TSH increased, adrenal insufficiency, thyroiditis, hypophysitis and diabetes mellitus), gastrointestinal irAEs (diarrhea and colitis), hepatic irAEs (alanine aminotransferase [ALT] increased, aspartate aminotransferase [AST] increased, amylase increased, transaminases increased, blood AP increased, Gamma Glutamyl Transpeptidase [GGT] increased, blood bilirubin increased and hepatitis), pulmonary irAEs (lung infection, pneumonitis, lung infiltration and interstitial lung disease) and renal irAEs (blood urea increased, blood creatinine increased, acute kidney failure, renal failure, acute renal failure and tubulointerstitial nephritis). If the ORRs evaluated by blind independent central review or investigators were both available, the former was preferred because of the low risk of detection bias [20]. Two researchers independently extracted the data. Any discrepancy was resolved by discussion.

### Statistical analysis

After logit transformation (logit(z) = log(z)-log(1-z)) on the incidence probability, we tested whether the transformed data are subject to normal distribution. The pooled estimates of the incidence rates of organ-classified irAEs were then generalized by package meta in R and the random model was used. The correlation between irAE incidence and ORR was tested by Pearson correlation test. Sensitivity analyses were performed by the exclusion of included trials by one cancer type each time. We set the nominal level of significance 5% and all 95% CIs were 2-sided. All statistical analyses were performed using GraphPad Prism 8 or R, version 3.6.0 (packages meta, R foundation).

## Results

### Eligible studies and characteristics

**Fig. 1:**
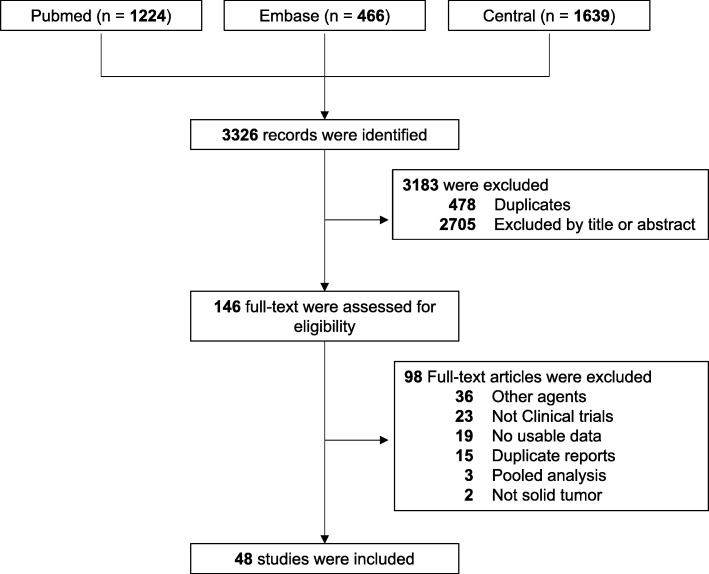
PRISMA flow diagram of the study selection

In total, 3326 records were identified via electronic searching. Of these, 478 duplicates and 2705 studies with irrelevant title and abstract were removed. With further reading of the full-text, 98 articles were excluded. The remaining 48 studies of 7936 patients with solid tumours treated with NIVO or NIVO+IPI were included in our meta-analysis. The procedure of the study selection is shown in Fig. 1.

The characteristics of the included studies are listed in Additional file 1: Table S1. There were 37 studies of 41 arms evaluating NIVO, and 19 studies of 25 arms evaluating NIVO+IPI, among which 8 studies evaluated both NIVO and NIVO+IPI. The trials involved the treatment of NSCLC (*n* = 13), melanoma (*n* = 12), renal clear cell carcinoma (*n* = 5), malignant pleural mesothelioma (*n* = 3), HNSCC (*n* = 3), urothelial carcinoma (*n* = 2), DNA mismatch repair-deficient or microsatellite instability-high colorectal cancer (*n* = 2), esophagogastric cancer (*n* = 2), anal cancer (*n* = 1), glioblastoma (*n* = 1), HCC (*n* = 1), ovarian cancer (*n* = 1), sarcoma (*n* = 1), and small cell lung cancer (*n* = 1). Of the included studies, more than half (30 of 48, 62.5%) reported both the incidence rates of select irAE (e.g., rash, pruritus) and the categorical incidence rates of a series of irAEs (e.g., skin).

### Incidence of immune-related adverse events

**Fig. 2:**
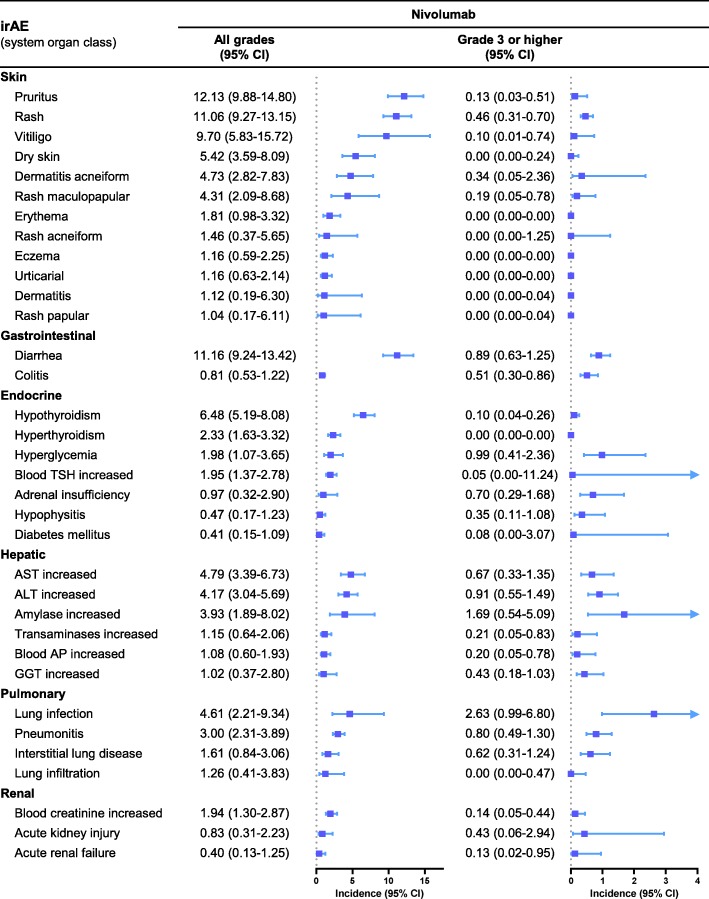
Incidence rates of the select immune-related adverse events of nivolumab. TSH, thyroid stimulating hormone; AST, aspartate aminotransferase; ALT, alanine aminotransferase; AP, alkaline phosphatase; GGT, γ-glutamyltransferase

**Fig. 3:**
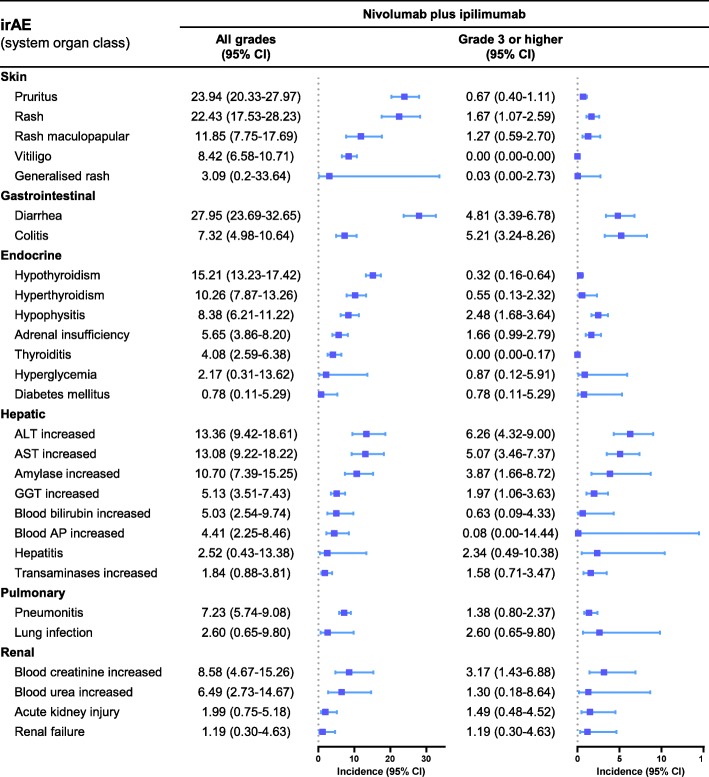
Incidence rates of the select immune-related adverse events of nivolumab plus ipilimumab. AST, aspartate aminotransferase; ALT, alanine aminotransferase; AP, alkaline phosphatase; GGT, γ-glutamyltransferase

We first analyzed the incidence rates for each select irAE. The irAE profiles for NIVO and NIVO+IPI are similar. The most common all grade irAEs were pruritus (12.13, 95% CI, 9.88–14.80%), diarrhea (11.16%; 95% CI, 9.24–13.42%) and rash (11.06%; 95% CI, 9.27–13.15%) for NIVO and diarrhea (27.95%; 323.69–32.65%), pruritus (23.94%; 95% CI, 20.33–27.97%) and rash (22.43%; 95% CI, 17.53–28.23%) for NIVO+IPI. The most common grade 3 or higher irAEs were lung infection (2.63%; 95% CI, 0.99–6.8%), amylase increased (1.69%; 95% CI, 0.54–5.09%) and hyperglycemia (0.99%; 95% CI, 0.41–2.36%) for NIVO and ALT increased (6.26%; 95% CI, 4.32–9.00%), colitis (5.21%; 95% CI, 3.24–8.26%) and AST increased (5.07%; 95% CI, 3.46–7.37%) for NIVO+IPI. The incidence rates of other irAEs are displayed in Figs. 2 and 3.

### Overall incidence of irAEs according to system organ class

IrAEs transpire in a large scale of organs, including skin, hepatic, renal, endocrine, gastrointestinal and pulmonary toxicities. The irAEs in different organs might derive from distinct autoantigens and manifest in varied severity and time of onset [3–8]. Here, we also summarize the incidence rates of irAEs according to system organ class. As for NIVO, the most common all-grade irAEs were skin (24.28%; 95% CI, 20.84–28.52%), gastrointestinal (10.73%; 95% CI, 8.85–12.97%) and endocrine toxicities (10.09%; 95% CI, 8.59–11.81%), and the most common grade 3 or higher irAEs were hepatic (1.26%; 95% CI, 10.84–1.89%), gastrointestinal (1.20%; 95% CI, 0.81–1.76%) and skin toxicities (0.99%; 95% CI, 0.66–1.49%) (Additional file 1: Table S2). For combination therapy of NIVO+IPI, the most common all-grade irAEs were skin (50.56%; 95% CI, 42.52–58.57%), gastrointestinal (33.55%; 95% CI, 27.18–40.58%) and endocrine toxicities (27.55%; 95% CI, 22.70–33.01%), and the most common grade 3 or higher irAEs were hepatic (10.06%; 95% CI, 7.12–14.03%), gastrointestinal (9.93%; 95% CI, 6.83–14.22%) and endocrine toxicities (4.07%; 95% CI, 3.03–5.43%) (Additional file 1: Table S2). Patients treated with NIVO+IPI had higher incidence rates of all grade and grade 3 or higher irAEs categorized by system organ class than NIVO (Additional file 1: Table S2).

### Correlation between the incidence of irAEs and ORR in patients treated with NIVO or NIVO+IPI

**Fig. 4:**
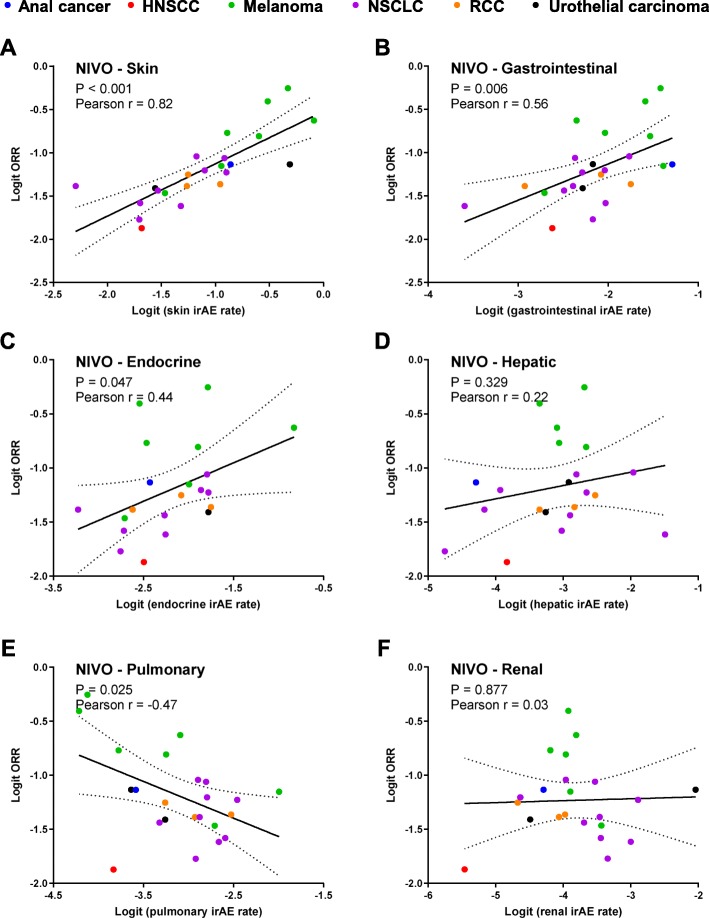
Correlation between response and immune-related adverse events by system organ class in nivolumab

The occurrence of irAE might be related to the enhancement of T cell-mediated immunoreaction, possibly indicating a better response to ICIs. Here, we investigated the correlation between ORR and the incidence rates of different irAEs in patients treated with NIVO or NIVO+IPI. For NIVO, 20 studies involving 23 arms reported both incidence rates of irAEs categorized by system organ class and ORR. The trials involved the treatment of NSCLC (*n* = 9), melanoma (*n* = 6), urothelial carcinoma (*n* = 2), RCC (*n* = 1), anal cancer (*n* = 1), and HNSCC (*n* = 1). As shown in Fig. 4, the ORR was positively correlated with the incidence rates of skin (r = 0.79, *P* < 0.001), gastrointestinal (r = 0.56, *P* = 0.006) and endocrine irAEs (r = 0.44, *P* = 0.05), while the ORR was negatively correlated with incidence of pulmonary irAEs (r = − 0.47, *P* = 0.02). These results suggested that the skin, gastrointestinal and endocrine irAEs might be positively associated with the clinical benefit of single-agent nivolumab, while the pulmonary irAEs might be negatively associated with the clinical benefit of NIVO.

**Fig. 5:**
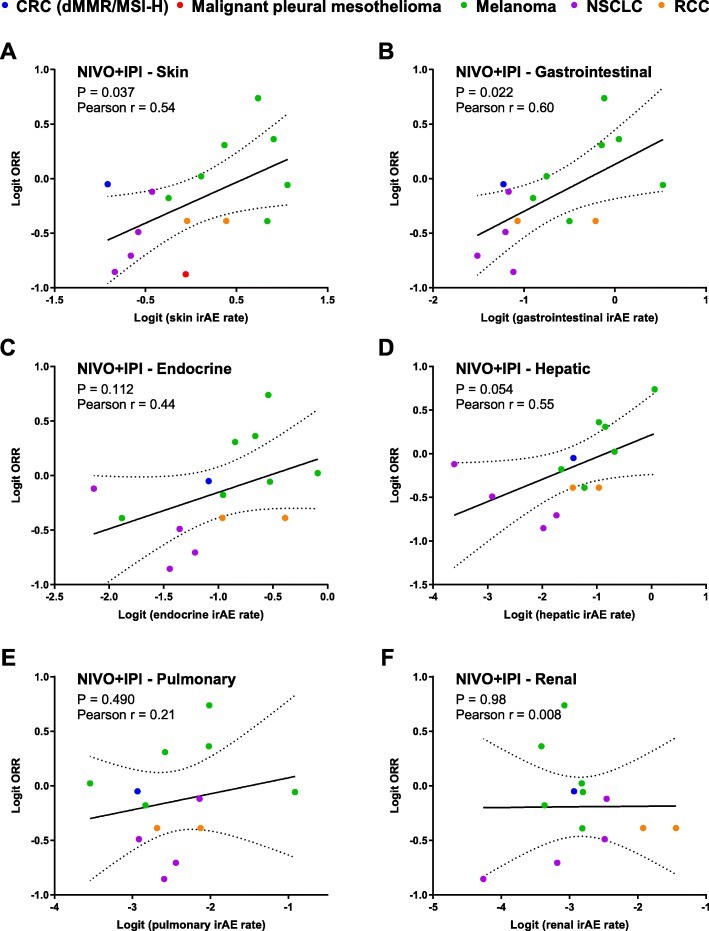
Correlation between response and immune-related adverse events by system organ class in nivolumab plus ipilimumab

For NIVO+IPI, 12 studies involving 15 arms with both incidence rates of irAEs categorized by system organ class and ORR were included in the analysis. The trials involved the treatment of melanoma (*n* = 6), NSCLC (*n* = 3), RCC (*n* = 1), CRC with dMMR-MSI-H (*n* = 1) and malignant pleural mesothelioma (*n* = 1). Consistent with the results in single-agent nivolumab, the ORR was positively correlated with skin (r = 0.54, *P* = 0.04) and gastrointestinal (r = 0.60, *P* = 0.02) (Fig. 5). Borderline significance was also observed in endocrine irAEs (r = 0.44, *P* = 0.11) and hepatic irAEs (r = 0.55, *P* = 0.05) (Fig. 5).

Apart from the arms of administrating NIVO or NIVO+IPI, 6 control arms undergoing chemotherapy from 6 trials were included to explore whether irAEs are relative to non-immunotherapeutic treatment. As a result, no correlation was observed between the incidence rates of irAEs and chemotherapeutic ORR (Additional file 1: Figure S1).

### Sensitivity analysis of the correlation between irAEs and ORR

In order to exclude the possibility that the correlations between the incidence rates of irAEs and ORR are mainly contributed by a single cancer type, we performed a sensitivity analysis by omitting studies of a cancer type at each time. For NIVO, the correlation between ORR and the incidence rates of skin, gastrointestinal or endocrine irAEs remained significant or borderline significant in all the sensitivity analysis (Additional file 1: Table S3), further confirming the clinical value of these irAEs. The negative correlation between ORR and incidence rates of pulmonary irAEs was not observed when studies of melanoma were excluded (Additional file 1: Table S3). While in NIVO+IPI, the correlation between ORR and the incidence rates of skin and gastrointestinal and hepatic irAEs were not significant when studies of NSCLC or melanoma were omitted (Additional file 1: Table S4), which may due to the limited numbers of included studies.

## Discussion

We completed the largest and most comprehensive meta-analysis to our knowledge concerning the incidence rates of irAEs induced by NIVO and NIVO+IPI, and firstly investigated their correlations with ORR in a pan-cancer setting. From the data of 48 trials including 7936 patients treated with NIVO or NIVO+IPI, we unmasked the positive associations between ORR and the incidence rates of skin and gastrointestinal irAEs. This meta-analysis provides an insight that skin and gastrointestinal irAEs might be potential indicators for the response to NIVO or NIVO+IPI.

This meta-analysis firstly summarizes the irAE profiles of NIVO and NIVO+IPI published to date. The irAE profiles of these two regimens are analogous, of which the most common all-grade irAEs occurred in skin, gastrointestinal and endocrine systems, and the most common grade 3 or higher irAEs took place in hepatic, gastrointestinal and skin systems. Despite this similarity in the relative order, the incidence rates of categorical irAEs were markedly higher when combined with ipilimumab. The deleterious effects of severe irAEs might outweigh the benefit from the addition of ipilimumab, which requires further evaluation on the cost-effective issue. As for clinical practice, close monitoring of the high-incidence irAEs might enable the early diagnosis and treatment, which thereby potentially reduce the usage of steroids and the irAE-related death.

In the present study, we elucidated the positive correlation between ORR and the incidence rates of skin, gastrointestinal and endocrine irAEs in NIVO and/or NIVO+IPI treatments in solid tumours. Autoimmune reactions contribute greatly to the induction of irAEs. Shared T-cell receptor repertoire was revealed in tumour and other tissues appearing irAE [21, 22], partially due to the tumour-tissue similarity [21]. In a recent study of NSCLC, skin ranked 2nd after lung itself and colon ranked 4th across multiple tissues in the tumour-tissue similarity score [21]. T-cells recognize shared antigens of tumour and skin and therefore target both organs, which is associated with the development of skin irAEs and likely with tumour regression as well [23]. Besides the identical T-cell clones within tumour and normal tissue, the mechanism underlying irAEs might include the pre-existing autoimmunity liberated after the successful blockade of immune checkpoint. NSCLC subjects with positive thyroid antibody at baseline before the initiation of ICIs are significantly at the risk of getting thyroid dysfunction as irAE [24, 25], and these preexisting antibodies are associated with better response to ICIs [24, 25]. Thus far, previous studies were almost implemented in NSCLC cohorts, while in our results, despite the omission of NSCLC trials, the correlation between the incidence rates of skin or gastrointestinal irAEs and ORR remained significant among other solid tumours. This result indicates that the principles for the association between irAEs and ORR in NSCLC patients discussed above, could be conceivably extended to other neoplasms. Future discoveries are needed to further elaborate the mechanism underlying this correlation.

Lead time bias critically contaminates the predictive efficacy of irAEs to better response to ICIs as patients who progress either switch to other therapies, while those who respond to immunotherapies have longer treatment duration and more time to develop autoimmune toxicities, especially in studies without a landmark design [23]. However, if all the occurrence of irAEs were on account of the longer period of immunotherapy, then we would expect the same positive correlation between ORR with the incidence rates of all kinds of irAEs, especially for those irAEs with low incidences. However, we didn’t observe the correlation between ORR and renal, hepatic and pulmonary toxicities. In addition, the median time of onset for skin and gastrointestinal irAEs was within 2 months [3–8], indicating that above half of these irAEs occurred before the first evaluation of response. Altogether, these enable skin and gastrointestinal irAEs to be a potential candidate for monitoring the response of NIVO or NIVO+IPI, even before the first CT scan.

We also observed a negative correlation between the incidence rates of pulmonary irAEs and ORR. According to a previous meta-analysis, immune-related pneumonitis accounted for 28.0% of death in clinical trials [9]. In severe cases of potentially fatal irAEs, systemic administration of steroid and discontinuation of ICI treatment will be performed [15, 16] and in most of the clinical trials, steroid use was allowed to manage irAEs. In a retrospective study of NSCLC patients receiving PD-1 checkpoint blockade, patients receiving > 10 mg/day of the steroid prednisone exhibited poorer outcomes (decreased PFS and OS) than patients taking < 10 mg/day [18]. These results might explain the negative correlation between the incidence rates of pulmonary irAEs and ORR in patients treated with NIVO. However, the incidence of steroid use was seldom reported, making it difficult to further analysis the interference of steroid use. The underlying mechanism needs to be further studied.

Our findings may provide important implications for the clinical practice in immunotherapy. Firstly, compared to NIVO, extensive irAEs were observed when combining with ipilimumab, which requires intensive monitoring to prevent the deterioration of lethal irAEs, e.g., pneumonia, and fulminant myocarditis. Secondly, our study indicates the existence of skin and gastrointestinal irAEs might be associated with favorable response to NIVO or NIVO+IPI. Early prediction of responses to NIVO or NIVO+IPI is of great clinical value. Compared with other irAEs, the skin and gastrointestinal irAEs may be used to predict the response to NIVO or NIVO+IPI in clinic.

Several limitations of this meta-analysis need to be stated. First, the possibility of lead time bias cannot be ruled out based on the data extracted from published articles, especially for those irAEs with low incidence rates. However, we didn’t observe the positive correlation between ORR and hepatic, pulmonary and renal irAEs. The median time of onset for skin and gastrointestinal irAEs is within 2 months in most trials [3–8]. The early-onset of these irAEs suggests that this correlation is not simply related to patients who remain on therapy longer being at greater risk to toxicity. Whether these two categories of irAEs can be applied to predict the response to NIVO and NIVO+IPI needs to be further studied with a land-mark analysis. Second, we only performed the meta-analysis in studies of NIVO or NIVO+IPI, where AEs were often reported according to system organ class. Clinical trials of other agents seldom reported irAEs categorized by system organ class, which make it hard to explore the correlation. Third, small-study effects might influence the correlation analysis when included studies with smaller sample sizes and relatively deviated irAE incidence rates. Forth, the number of clinical trials for NIVO+IPI was relatively limited. The correlation between irAEs and ORR was no longer significant if studies of melanoma (*n* = 9) or NSCLC (*n* = 4) were omitted. More clinical trials are needed to study the correlation between irAEs and ORR of NIVO+IPI.

## Conclusions

This is the first meta-analysis to our knowledge to summarize the incidence rates of irAEs in patients with advanced solid tumours treated with NIVO or NIVO+IPI. The detriment of severe irAEs from the addition of ipilimumab might outweigh the benefit, which requires further comparative studies on the cost-effectiveness. Following correlation analysis uncovers the association between irAEs and ORR in NIVO and/or NIVO+IPI trials across multiple neoplasms, which highlights the potential of predictive value of irAEs to favorable response of immunotherapy. Such an understanding might help in auxiliary distinguishing pseudo-progression and determining whether resuming immunotherapy after the recovery from manageable irAEs, thereby fostering the clinical application of ICIs in patients with advanced solid tumours.

## Supplementary information

**Additional file 1: Method.** Search strategy. **Table S1.** Characteristics of the included studies. **Table S2.** Incidences of Categorical irAEs according to system organ class. **Table S3.** Sensitivity analysis of the correlation between irAEs and ORR in NIVO. **Table S4.** Sensitivity analysis of the correlation between irAEs and ORR in NIVO+IPI. **Figure S1.** Correlation between irAEs and ORR of chemotherapy-contained regimen. **References (DOCX 276 kb)**

## Data Availability

All data generated or analyzed during this study are included in the published article.

## Abbreviations

ALT: Alanine aminotransferase
AST: Aspartate aminotransferase
CRC: Colorectal carcinoma
CTLA-4: Cytotoxic T lymphocyte associated antigen 4
GGT: Gamma Glutamyl Transpeptidase
HCC: Hepatocellular carcinoma
HNSCC: Head and neck squamous cell carcinoma
irAE: Immune-related adverse event
MeSH: Medical subject headings
NIVO: Nivolumab
NIVO+IPI: Nivolumab plus ipilimumab
NSCLC: Non-small cell lung cancer
ORR: Objective response rates
OS: Overall survival
PD-1: Programmed cell death-1
PD-L1: Programmed cell death-ligand 1
PFS: Progressive-free survival
PRISMA: Preferred Reporting Items for Systematic reviews and Meta-Analyses
RCC: Renal cell carcinoma
