# Correction: *Incidence rates of immune-related adverse events and their correlation with response in advanced solid tumours treated with NIVO or NIVO+IPI: a systematic review and meta-analysis*

**DOI:** 10.1136/jitc-2020-0779-6corr1

**Published:** 2020-07-16

Xing P, Zhang F, Wang G, *et al.* Incidence rates of immune-related adverse events and their correlation with response in advanced solid tumours treated with NIVO or NIVO+IPI: a systematic review and meta-analysis. *J ImmunoTher Cancer* 2019;7:341. doi: 10.1186/s40425-019-0779-6

In the article titled ‘Incidence rates of immune-related adverse events and their correlation with response in advanced solid tumours treated with NIVO or NIVO+IPI: a systematic review and meta-analysis’, the author noticed that the second corresponding author was not designated in the article.

The correct corresponding authors are:

Junling Li - Department of Medical Oncology, National Cancer Center/National Clinical Research Center for Cancer/Cancer Hospital, Chinese Academy of Medical Sciences and Peking Union Medical College, Beijing, China, 17 Pan-jia-yuan South Lane, Chaoyang District, 100021, Beijing, China. Email: lijunling@cicams.ac.cn

Yan Wang - Department of Medical Oncology, National Cancer Center/National Clinical Research Center for Cancer/Cancer Hospital, Chinese Academy of Medical Sciences and Peking Union Medical College, Beijing, China, 17 Pan-jia-yuan South Lane, Chaoyang District, 100021, Beijing, China. Email: wangyanyifu@126.com

