# Supplementary material for: Incidence rates of immune-related adverse events and their correlation with response in advanced solid tumours treated with NIVO or NIVO+IPI: a systematic review and meta-analysis
Source: J Immunother Cancer. 2019 Dec 4;7:341. doi: 10.1186/s40425-019-0779-6 (PMC6894272; doi:10.1186/s40425-019-0779-6)
Supplement: Supplementary file 1 — Additional file 1: Method. Search strategy. Table S1. Characteristics of the included studies. Table S2. Incidences of Categorical irAEs according to system organ class. Table S3. Sensitivity analysis of the correlation between irAEs and ORR in NIVO. Table S4. Sensitivity analysis of the correlation between irAEs and ORR in NIVO+IPI. Figure S1. Correlation between irAEs and ORR of chemotherapy-contained regimen. References (DOCX 276 kb) [file 40425_2019_779_MOESM1_ESM.docx]

**Supplementary Online content**

Xing P et al. Incidence rates of immune-related adverse events and their correlation with response in advanced solid tumours treated with NIVO or NIVO+IPI: a systematic review and meta-analysis

**Supplemental Method.** Search strategy.

**Supplemental Table 1.** Characteristics of the included studies.

**Supplemental Table 2.** Incidences of Categorical irAEs according to system organ class.

**Supplemental Table 3.** Sensitivity analysis of the correlation between irAEs and ORR in NIVO.

**Supplemental Table 4.** Sensitivity analysis of the correlation between irAEs and ORR in NIVO+IPI.

**Supplemental Figure 1.** Correlation between irAEs and ORR of chemotherapy-contained regimen.

**Supplemental References**

This supplementary material has been provided by the authors to give readers additional information about their work.

**Supplemental Method. Search strategy.**

**Embase**

#1 ‘nivolumab’/exp

#2 ‘nivolumab’

#3 ‘ipilimumab’

#4 #1 **OR** #2 **OR** #3

#5 #4 **AND** ([article]/lim **OR** [article in press]/lim)

#6 #5 **AND** [clinical trial number]/lim **AND** [humans]/limit **AND** [clinical study]/lim

#7 #6 **AND** [1-1-2015]/sd **NOT** [1-5-2019]/sd

**PubMed**

#1 nivolumab

#2 ipilimumab

#3 clinical trials as topic [Mesh] **OR** (clinical **AND** trial **AND** topic) **OR** clinical trials as topic **OR** trial

#4 #2 **AND** #3

#5 Animals [Mesh] **NOT** human [Mesh]

#6 #4 **NOT** #5

#7 #6 **AND** (“2000/01/01”[Date-Publication] : “2019/05/01”[Date-Publication])

**Central Register of Controlled Trials of the Cochrane Library**

#1 nivolumab

#2 ipilimumab

#3 #1 **OR** #2 in trial

**Supplemental Table 1. Characteristics of the included studies.**

| **Study** | **Trial ID** | **NCT** | **Type of tumour** | **Phase** | **Treatment** | **Dosing schedule** | **No. of patient** | **AE report criteria** | **Evaluator** | **Criteria for ORR** | **ORR** |
| --- | --- | --- | --- | --- | --- | --- | --- | --- | --- | --- | --- |
| 2014-Topalian-J Clin Oncol^1^ | CA209-003 | NCT00730639 | Melanoma | NA | NIVO | 0.1, 0.3, 1.0 mg/kg q2w | 107 | Treatment-related; ≥3% of patients | Investigator | RECIST v1.0 with modification | 31% |
| 2015-Borghaei-N Engl J Med^2^ | CheckMate 057 | NCT01673867 | NSCLC | III | NIVO | 3 mg/kg, q2w | 292 | Treatment-related; ≥10% of patients; select TRAE | Investigator | RECIST v1.1 | 19% |
|  |  |  |  |  | Docetaxel | 75 mg/m^2^, q3w | 290 | Treatment-related; ≥10% of patients; select TRAE | Investigator | RECIST v1.1 | 12% |
| 2015-Brahmer-N Engl J Med^3^ | CheckMate 017 | NCT01642004 | NSCLC | III | NIVO | 3 mg/kg, q2w | 135 | Treatment-related; ≥5% of patients; select TRAE | Investigator | RECIST v1.1 | 20% |
|  |  |  |  |  | Docetaxel | 75 mg/m^2^, q3w | 137 | Treatment-related; ≥5% of patients; select TRAE | Investigator | RECIST v1.1 | 9% |
| 2015-McDermott-J Clin Oncol^4^ |  | NCT0730639 | RCC | I | NIVO | 1, 10 mg/kg q2w | 34 | Treatment-related; ≥3% of patients | Investigator | RECIST v1.0 with modification | 29% |
| 2015-Hamanishi-J Clin Oncol^5^ |  | UMIN000005714 | Ovarian cancer | II | NIVO | 1, 3 mg/kg q2w | 20 | Treatment-related; ≥20% of patients | BICR | RECIST v1.1 | 15% |
| 2015-Larkin-N Engl J Med^6^ | CheckMate 067 | NCT01844505 | Melanoma | III | NIVO | 3 mg/kg, q2w | 316 | Treatment-related; ≥5% of patients | Investigator | RECIST v1.1 | 44% |
|  |  |  |  |  | NIVO+IPI | NIVO 1 mg/kg, IPI 3 mg/kg, q3w for 4 cycles; then NIVO 3 mg/kg q2w | 314 | Treatment-related; ≥5% of patients | Investigator | RECIST v1.1 | 58% |
| 2015-Postow-N Engl J Med^7^ | Checkmate 069 | NCT01927419 | Melanoma | I | NIVO+IPI | NIVO 1 mg/kg, IPI 3 mg/kg, q3w for 4 cycles; then NIVO 3 mg/kg q2w | 95 | Treatment-related; ≥10% of patients; select TRAE | Investigator | RECIST v1.1 | 59% |
| 2015-Rizvi-Lancet Oncol^8^ | CheckMate 063 | NCT01721759 | NSCLC | II | NIVO | 3 mg/kg, q2w | 117 | Treatment-related; ≥5% of patients; immune-mediated TRAE | BICR | RECIST v1.1 | 15% |
| 2015-Motzer-N Engl J Med^9^ | CheckMate 025 | NCT01668784 | RCC | III | NIVO | 3 mg/kg, q2w | 410 | Treatment-related; ≥10% of patients | Investigator | RECIST v1.1 | 25% |
| 2015-Motzer-J Clin Oncol^10^ | NA | NCT01354431 | RCC | II | NIVO | 0.3 mg/kg, q3w | 60 | Treatment-related; ≥10% of patients; select TRAE | Investigator | RECIST v1.1 | 20% |
|  |  |  |  |  | NIVO | 2 mg/kg, q3w | 54 | Treatment-related; ≥10% of patients; select TRAE | Investigator | RECIST v1.1 | 22% |
|  |  |  |  |  | NIVO | 10 mg/kg, q3w | 54 | Treatment-related; ≥10% of patients; select TRAE | Investigator | RECIST v1.1 | 20% |
| 2015-Robert-N Engl J Med^11^ | CheckMate 066 | NCT01721772 | Melanoma | III | NIVO | 3 mg/kg, q2w | 210 | Treatment-related; ≥10% of patients; select TRAE of potential immunological etiology | Investigator | RECIST v1.1 | 40% |
| 2015-Gettinger-J Clin Oncol^12^ | CA209-003 | NCT00730639 | NSCLC | I | NIVO | 1 3 10 mg/kg | 129 | Treatment-related; ≥3% of patients | Not mentioned | RECIST v1.0 | 17% |
| 2015-Weber-Lancet Oncol^13^ | CheckMate 037 | NCT01721746 | Melanoma | III | NIVO | 3 mg/kg, q2w | 272 | Treatment-related; ≥5% of patients; select TRAE of potential immune-related | Investigator | RECIST v1.1 | 32% |
|  |  |  |  |  | Dacarbazine or chemo | dacarbazine 1000 mg/m², q3w, or carboplatin area under the curve 6 plus paclitaxel 175 mg/m² , q3w | 133 | Treatment-related; ≥5% of patients; select TRAE of potential immunological etiology | Investigator | RECIST v1.1 | 11% |
| 2016-Weber-Lancet Oncol^14^ | CheckMate 064 | NCT01783938 | Melanoma | II | NIVO+IPI | NIVO 3mg/kg q2w 6 cycles; then IPI 3mg/kg q3w; then NIVO 3 mg/kg q2w | 68 | Treatment-related; ≥5% of patients; select TRAE of potential immunological etiology | Investigator | RECIST v1.1 | 68% |
| 2016-Weber-Cancer Immunol Res^15^ | NA | NCT01176461 | Melanoma | I/II | NIVO | 3 mg/kg q2w | 92 | Treatment-related; >5% of patients | Not mentioned | mWHO and immune-related criteria | 29% |
| 2016-Nishio-ESMO open^16^ | NA | JapicCTI-132073 | NSCLC | II | NIVO | 3 mg/kg q2w | 76 | Treatment-related; ≥5% of patients; select treatment-related AE | BICR | RECIST v1.1 | 23% |
| 2016-Sharma-Lancet Oncol^17^ | CheckMate 032 | NCT01928394 | Urothelial carcinoma | I/II | NIVO | 3 mg/kg q2w | 78 | Treatment-related; ≥10 patients or grade 3/4 | Investigator | RECIST v1.1 | 24% |
| 2016-Gettinger-J Clin Oncol^18^ | CheckMate 012 | NCT01454102 | NSCLC | I | NIVO | 3 mg/kg q3w | 52 | Treatment-related; ≥5% of patients; TRAE | Investigator | RECIST v1.1 | 23% |
| 2016-Antonia-Lancet Oncol^19^ | CheckMate 032 | NCT01928394 | SCLC | I/II | NIVO | 3 mg/kg, q2w | 98 | Treatment-related; ≥10% of patients or grade 3/4 | Investigator | RECIST v1.1 | 10% |
|  |  |  |  |  | NIVO+IPI | NIVO 1 mg/kg, IPI 3 mg/kg, q3w for 4 cycles; then NIVO 3 mg/kg q2w | 61 | Treatment-related; ≥10% of patients or grade 3/4 | Investigator | RECIST v1.1 | 23% |
|  |  |  |  |  | NIVO+IPI | NIVO 3 mg/kg, IPI 1 mg/kg, q3w for 4 cycles; then NIVO 3 mg/kg q2w | 54 | Treatment-related; ≥10% of patients or grade 3/4 | Investigator | RECIST v1.1 | 19% |
| 2017-El-Khoueiry-Lancet Oncol^20^ | CheckMate 040 | NCT01658878 | HCC | I/II | NIVO | does-escalation | 48 | Treatment-related; ≥5% of patients | Investigator | RECIST v1.1 | 15% |
|  |  |  |  |  | NIVO | does-expansion | 214 | Treatment-related; ≥5% of patients | Investigator | RECIST v1.1 | 20% |
| 2017-Carbone-N Engl J Med^21^ | CheckMate 026 | NCT02041533 | NSCLC | III | NIVO | 3 mg/kg, q2w | 271 | Treatment-related; ≥10% of patients; select TRAE | BICR | RECIST v1.1 | 26% |
|  |  |  |  |  | Chemo | administered once every 3 weeks for up to six cycles | 270 | Treatment-related; ≥10% of patients; select TRAE | BICR | RECIST v1.1 | 33% |
| 2017-Hammers-J Clin Oncol^22^ | checkmate 016 | NCT01472081 | RCC | I | NIVO+IPI | NIVO 3 mg/kg q2w, IPI 1 mg/kg q3w; then NIVO 3 mg/kg q2w | 47 | Treatment-related; ≥20% of patients or ≥ 2 patients for grade 3/4; select TRAE | Investigator | RECIST v1.1 | 40% |
|  |  |  |  |  | NIVO+IPI | NIVO 1 mg/kg q2w，IPI 3 mg/kg q3w; then NIVO 3 mg/kg q2w | 47 | Treatment-related; ≥20% of patients or ≥ 2 patients for grade 3/4; select TRAE | Investigator | RECIST v1.1 | 40% |
| 2017-Hellmann-Lancet Oncol^23^ | CheckMate 012 | NCT01454102 | NSCLC | I | NIVO+IPI | NIVO 3 mg/kg, q2w; IPI 1 mg/kg, q12w | 38 | Treatment-related; ≥10% of patients or grade 3/4; select TRAE | Investigator | RECIST v1.1 | 47% |
|  |  |  |  |  | NIVO+IPI | NIVO 3 mg/kg, q2w; IPI 1 mg/kg, q6w | 40 | Treatment-related; ≥10% of patients or grade 3/4; select TRAE | Investigator | RECIST v1.1 | 38% |
| 2017-Overman-Lancet Oncol^24^ | CheckMate 142 | NCT02060188 | CRC (dMMR/MSI-H) | II | NIVO | 3 mg/kg, q2w | 74 | Treatment-related; ≥10% of patients or grade 3/4 | BICR | RECIST v1.1 | 36% |
| 2017-Yamazaki-Cancer Sci^25^ | NA | JapicCTI-142533 | Melanoma | II | NIVO | 3 mg/kg q2w | 24 | Treatment-related; ≥10% of patients; select TRAE | BICR | RECIST v1.1 | 35% |
| 2017-Sharma-Lancet Oncol^26^ | CheckMate 275 | NCT02387996 | Urothelial carcinoma | II | NIVO | 3 mg/kg, q2w | 270 | Treatment-related; ≥10% of patients; select TRAE | BICR | RECIST v1.1 | 20% |
| 2017-Kudo-Lancet Oncol^27^ | ATTRACTION01 | JapicCTI-142422 | Oesophageal squamous-cell | II | NIVO | 3 mg/kg, q2w | 65 | Treatment-related; ≥10% of patients or grade 3/4 | BICR | RECIST v1.1 | 22% |
| 2017-Hida-Cancer Sci^28^ | NA | JapicCTI-132072 | NSCLC | II | NIVO | 3 mg/kg, q2w | 35 | Treatment-related; ≥ 5% of patients; select TRAE | BICR | RECIST v1.1 | 26% |
| 2017-Morris-Lancet Oncol^29^ | NCI9673 | NCT02314169 | Anal cancer | II | NIVO | 3 mg/kg q2w | 37 | All AEs | Investigator | RECIST v1.1 | 24% |
| 2017-Kang-Lancet^30^ | ATTRACTION02 | NCT02267343 | Gastric or gastro-oesophageal junction | III | NIVO | 3 mg/kg, q2w | 330 | Treatment-related; ≥2% of patients | Investigator | RECIST v1.1 | 11% |
| 2018-Omuro-Neuro Oncol^31^ | CheckMate 143 | NCT02017717 | Glioblastoma | I | NIVO | 3 mg/kg q2w | 10 | Treatment-related; ≥ 2 patients or grade 3/4 | Investigator | RANO criteria | 11% |
|  |  |  |  |  | NIVO+IPI | NIVO 1 mg/kg q2w, IPI 3 mg/kg q3w; then NIVO 3 mg/kg q2w | 10 | Treatment-related; ≥ 2 patients or grade 3/4 | Investigator | RANO criteria | 0% |
|  |  |  |  |  | NIVO+IPI | NIVO 1 mg/kg q2w, IPI 3 mg/kg q3w; then NIVO 3 mg/kg q2w | 20 | Treatment-related; ≥ 2 patients or grade 3/4 | Investigator | RANO criteria | 10% |
| 2018-Ma-J Clin Oncol^32^ | NCI-9742 | NCT02339558 | Nasopharyngeal Carcinoma | II | NIVO | 3 mg/kg q2w | 45 | Select treatment-related AE | Not mentioned | RECIST v1.1 | 20% |
| 2018-Long-Lancet Oncol^33^ |  | NCT02374242 | Melanoma with brain metastasis | II | NIVO+IPI | NIVO 1 mg/kg, IPI 3 mg/kg, q3w; then NIVO 3 mg/kg q2w | 35 | Treatment-related; ≥10% of patients or grade 3/4; select TRAE | Investigator | RECIST v1.1 | 49% |
|  |  |  |  |  | NIVO | 3 mg/kg, q2w | 25 | Treatment-related; ≥10% of patients or grade 3/4; select TRAE | Investigator | RECIST v1.1 | 24% |
|  |  |  |  |  | NIVO | 3 mg/kg, q2w | 16 | Treatment-related; ≥10% of patients or grade 3/4; select TRAE | Investigator | RECIST v1.1 | 19% |
| 2018-Tawbi-N Engl J Med^34^ | CheckMate 204 | NCT02320058 | Melanoma with brain metastasis | II | NIVO+IPI | NIVO 1 mg/kg, IPI 3 mg/kg, q3w; then NIVO 3 mg/kg q2w | 94 | Treatment-related; ≥5% of patients or grade 3/4 | Investigator | RECIST v1.1 | 50% |
| 2013-Wolchok-N Engl J Med^35^ | NA | NCT01024231 | Melanoma | I | NIVO+IPI |  | 53 | Select treatment-related AE | Investigator | mWHO and immune-related criteria | 40% |
| 2018-Lee-Lung Cancer^36^ | NA | NCT02175017 | NSCLC | II | NIVO | 3 mg/kg q2w | 100 | Treatment-related; ≥10 of patients or grade 3/4 | BICR | RECIST v1.1 | 20% |
| 2018-Quispel-Janssen-J Thorac Oncol^37^ | NA | NCT02497508 | Malignant pleural mesothelioma | II | NIVO | 3 mg/kg q2w | 34 | Treatment-related | Not mentioned | RECIST with modification | 24% |
| 2018-Hellmann-N Engl J Med^38^ | CheckMate 227 | NCT02477826 | NSCLC | III | NIVO+IPI | NIVO 3 mg/kg, q2w; IPI 1 mg/kg, q6w | 583 | Treatment-related; ≥10% of patients | BICR | RECIST v1.1 | 33% |
|  |  |  |  |  | NIVO | 3 mg/kg, q2w | 396 | Treatment-related; ≥10% of patients | BICR | RECIST v1.1 |  |
| 2018-Overman-J Clin Oncol^39^ | CheckMate 142 | NCT02060188 | CRC (dMMR/MSI-H) | II | NIVO+IPI | nivo 3 mg/kg, ipi 1 mg/kg, q3w | 119 | Treatment-related; >10% of patients; select TRAE | BICR | RECIST v1.1 | 49% |
| 2018-Motzer-N Engl J Med^40^ | CheckMate 214 | NCT02231749 | RCC | III | NIVO+IPI | NIVO 3 mg/kg, IPI 1 mg/kg, q3w; then NIVO 3 mg/kg q2w | 425 | Treatment-related; ≥15% of patients | BICR | RECIST v1.1 | 42% |
| 2018-D'Angelo-Lancet Oncol^41^ | Alliance A091401 | NCT02500797 | Sarcoma | II | NIVO | 3 mg/kg, q2w | 43 | Treatment-related; ≥10% of patients or grade 3/4/5 | Investigator | RECIST v1.1 | 16% |
|  |  |  |  |  | NIVO+IPI | NIVO 3 mg/kg, IPI 1 mg/kg, q3w | 42 | Treatment-related; ≥10% of patients or grade 3/4/5 | Investigator | RECIST v1.1 | 7% |
| 2018-Janjigian-J Clin Oncol^42^ | CheckMate 032 | NCT01928394 | Esophagogastric | III | NIVO | 3 mg/kg, q2w | 59 | Treatment-related; ≥15% of patients | BICR | RECIST v1.1 | 7% |
|  |  |  |  |  | NIVO+IPI | NIVO 1 mg/kg, IPI 3 mg/kg, q3w for 4 cycles; then NIVO 3 mg/kg q2w | 49 | Treatment-related; ≥15% of patients | BICR | RECIST v1.1 | 20% |
|  |  |  |  |  | NIVO+IPI | NIVO 3 mg/kg, IPI 1 mg/kg, q3w for 4 cycles; then NIVO 3 mg/kg q2w | 52 | Treatment-related; ≥15% of patients | BICR | RECIST v1.1 | 4% |
| 2019-Wu-J Thorac Oncol^43^ | CheckMate 078 | NCT02613507 | NSCLC | III | NIVO | 3 mg/kg, q2w | 338 | Treatment-related; ≥10% of patients; select TRAE | Investigator | RECIST v1.1 | 17% |
|  |  |  |  |  | Docetaxel |  | 166 | Treatment-related; ≥10% of patients; select TRAE | Investigator | RECIST v1.1 | 4% |
| 2019-Lebbe-J Clin Oncol^44^ | Checkmate 511 | NCT02714218 | melanoma | IIIb/IV | NIVO+IPI | NIVO 3 mg/kg, IPI 1 mg/kg, q3w; then NIVO 480 mg q4w | 180 | Treatment-related; ≥10% of patients; select TRAE ≥5% of patients | Investigator | RECIST v1.1 | 46% |
|  |  |  |  |  | NIVO+IPI | NIVO 1 mg/kg, IPI 3 mg/kg, q3w; then NIVO 480 mg q4w | 178 | Treatment-related; ≥10% of patients; select TRAE ≥5% of patients | Investigator | RECIST v1.1 | 51% |
| 2019-Disselhorst-Lancet Respir Med^45^ | INITIATE | NCT03048474 | Malignant pleural mesothelioma | II | NIVO+IPI | NIVO 240 mg q2w, IPI 1 mg/kg q6w; then NIVO 240 mg q2w | 35 | Treatment-related; ≥ 10% of patients or grade 3/4 | BICR | mRECIST | 29% |
| 2019-Neal Ready-J Clin Oncol^46^ | CheckMate 568 | NCT02659059 | NSCLC | II | NIVO+IPI | NIVO 3 mg/kg q2w, IPI 1 mg/kg q6w | 288 | Treatment-related; ≥ 10% of patients; select TRAE | Investigator | RECIST v1.1 | 30% |
| 2019-Ferris-N Engl J Med^47^ | CheckMate 141 | NCT02105636 | HNSCC | III | NIVO | 3 mg/kg, q2w | 240 | Treatment-related; ≥5% of patients; select TRAE | Investigator | RECIST v1.1 | 13% |
|  |  |  |  |  | Methotrexate, docetaxel, or cetuximab |  | 121 | Treatment-related; ≥5% of patients; select TRAE | Investigator | RECIST v1.1 | 6% |
| 2019-Scherpereel-Lancet Oncol^48^ | IFCT-1501 MAPS2 | NCT02716272 | Malignant pleural mesothelioma | II | NIVO | 3 mg/kg q2w | 63 | Treatment-related; ≥10 of patients or grade 3/4 | BICR | RECIST v1.0 | 18% |
|  |  |  |  |  | NIVO+IPI | NIVO 3 mg/kg q2w, IPI 1 mg/kg q6w | 62 | Treatment-related; ≥10 of patients or grade 3/4 | BICR | RECIST v1.0 | 26% |

AE=adverse event. ORR=objective response rate. TRAE=treatment-related adverse event. BICR=Blinded independent central review. NIVO=nivolumab. IPI=ipilimumab. NIVO+IPI=nivolumab+ipilimumab. RECIST=response evaluation criteria in solid tumours. NSCLC=non-small cell lung cancer. RCC=renal cell carcinoma. HCC=hepatocellular carcinoma. CRC=colorectal carcinoma. HNSCC=head and neck squamous cell carcinoma.

**Supplemental Table 2. Incidences of Categorical irAEs according to system organ class.**

|  | **All grades** | | | |  | | **Grade 3 or higher** | | | |
| --- | --- | --- | --- | --- | --- | --- | --- | --- | --- | --- |
|  | **NIVO**  **(95% CI)** | **NIVO+IPI**  **(95% CI)** | **OR**  **(95% CI)** | **P** | |  | **NIVO**  **(95% CI)** | **NIVO+IPI**  **(95% CI)** | **OR**  **(95% CI)** | **P** |
| Skin | 24.48 (20.84-28.52) | 50.56 (42.52-58.57) | 0.44 (0.39-0.49) | <0.001 | |  | 0.99 (0.66-1.49) | 3.47 (2.23-5.36) | 0.28 (0.18-0.42) | <0.001 |
| Gastrointestinal | 10.73 (8.85-12.97) | 33.55 (27.18-40.58) | 0.32 (0.28-0.36) | <0.001 | |  | 1.20 (0.81-1.76) | 9.93 (6.83-14.22) | 0.13 (0.09-0.17) | <0.001 |
| Endocrine | 10.09 (8.59-11.81) | 27.55 (22.70-33.01) | 0.33 (0.28-0.38) | <0.001 | |  | 0.41 (0.19-0.88) | 4.07 (3.03-5.43) | 0.13 (0.08-0.23) | <0.001 |
| Hepatic | 4.87 (3.53-6.69) | 20.10 (14.39-27.35) | 0.28 (0.24-0.33) | <0.001 | |  | 1.26 (0.84-1.89) | 10.06 (7.12-14.03) | 0.12 (0.09-0.16) | <0.001 |
| Pulmonary | 4.23 (3.38-5.28) | 7.77 (5.83-10.29) | 0.54 (0.43-0.67) | <0.001 | |  | 0.91 (0.52-1.59) | 1.66 (1.11-2.46) | 0.61 (0.36-1.04) | 0.07 |
| Renal | 1.98 (1.34-2.93) | 5.14 (3.51-7.47) | 0.46 (0.34-0.64) | <0.001 | |  | 0.22 (0.10-0.48) | 1.65 (0.99-2.75) | 0.14 (0.06-0.33) | <0.001 |

NIVO=nivolumab. NIVO+IPI=nivolumab+ipilimumab. OR=odds ratio. CI=confidence interval

**Supplemental Table 3. Sensitivity analysis of the correlation between irAEs and ORR in NIVO**

| Omitted studies | No. of  omitted arms | Skin irAEs | |  | Gastrointestinal irAEs | |  | Endocrine irAEs | |  | Hepatic irAEs | |  | Pulmonary irAEs | |  | Renal irAEs | |
| --- | --- | --- | --- | --- | --- | --- | --- | --- | --- | --- | --- | --- | --- | --- | --- | --- | --- | --- |
|  |  | P | r |  | P | r |  | P | r |  | P | r |  | P | r |  | P | r |
| Anal cancer | 1 | <0.001 | 0.79 |  | 0.004 | 0.59 |  | 0.05 | 0.44 |  | 0.29 | 0.25 |  | 0.03 | -0.47 |  | 0.85 | 0.04 |
| HNSCC | 1 | <0.001 | 0.78 |  | 0.01 | 0.54 |  | 0.06 | 0.42 |  | 0.50 | 0.16 |  | 0.001 | -0.64 |  | 0.34 | -0.22 |
| Melanoma | 7 | 0.009 | 0.63 |  | 0.07 | 0.47 |  | 0.08 | 0.48 |  | 0.31 | 0.27 |  | 0.74 | 0.09 |  | 0.45 | 0.20 |
| NSCLC | 9 | <0.001 | 0.83 |  | 0.04 | 0.56 |  | 0.26 | 0.34 |  | 0.26 | 0.35 |  | 0.13 | -0.43 |  | 0.27 | 0.33 |
| RCC | 3 | <0.001 | 0.80 |  | 0.007 | 0.58 |  | 0.06 | 0.46 |  | 0.33 | 0.24 |  | 0.04 | -0.46 |  | 0.94 | 0.02 |
| Urothelial carcinoma | 2 | <0.001 | 0.82 |  | 0.009 | 0.56 |  | 0.04 | 0.47 |  | 0.36 | 0.22 |  | 0.03 | -0.48 |  | 0.92 | -0.02 |

irAE=immune-related adverse event. ORR=objective response rate. HNSCC=head and neck squamous cell carcinoma. NSCLC=non-small cell lung cancer. RCC=renal cell carcinoma.

**Supplemental Table 4. Sensitivity analysis of the correlation between irAEs and ORR in NIVO+IPI**

| Omitted studies | No. of  omitted arms | Skin irAEs | |  | Gastrointestinal irAEs | |  | Endocrine irAEs | |  | Hepatic irAEs | |  | Pulmonary irAEs | |  | Renal irAEs | |
| --- | --- | --- | --- | --- | --- | --- | --- | --- | --- | --- | --- | --- | --- | --- | --- | --- | --- | --- |
|  |  | P | r |  | P | r |  | P | r |  | P | r |  | P | r |  | P | r |
| CRC (dMMR/MSI-H) | 1 | 0.01 | 0.64 |  | 0.02 | 0.65 |  | 0.12 | 0.45 |  | 0.07 | 0.55 |  | 0.47 | 0.23 |  | 0.97 | 0.01 |
| Malignant pleural mesothelioma | 1 | 0.03 | 0.57 |  | 0.02 | 0.60 |  | 0.11 | 0.44 |  | 0.05 | 0.55 |  | 0.49 | 0.21 |  | 0.97 | 0.01 |
| Melanoma | 7 | 0.93 | -0.04 |  | 0.77 | 0.14 |  | 0.84 | -0.10 |  | 0.75 | -0.15 |  | 0.99 | 0.01 |  | 0.21 | 0.54 |
| NSCLC | 4 | 0.32 | 0.33 |  | 0.25 | 0.40 |  | 0.31 | 0.36 |  | 0.02 | 0.76 |  | 0.67 | 0.17 |  | 0.10 | 0.59 |
| RCC | 2 | 0.04 | 0.57 |  | 0.02 | 0.65 |  | 0.07 | 0.54 |  | 0.05 | 0.60 |  | 0.50 | 0.23 |  | 0.47 | 0.25 |

irAE=immune-related adverse event. ORR=objective response rate. dMMR/MSI-H=mismatch repair deficient/micro-satellite instability high. NSCLC=non-small cell lung cancer. RCC=renal cell carcinoma.

**Supplemental Figure 1. Correlation between irAEs and ORR of chemotherapy-contained regimen.** The correlation between ORR of chemotherapy-contained regimen and irAEs categorized by system organ class (A-F). HNSCC=head and neck squamous cell carcinoma.


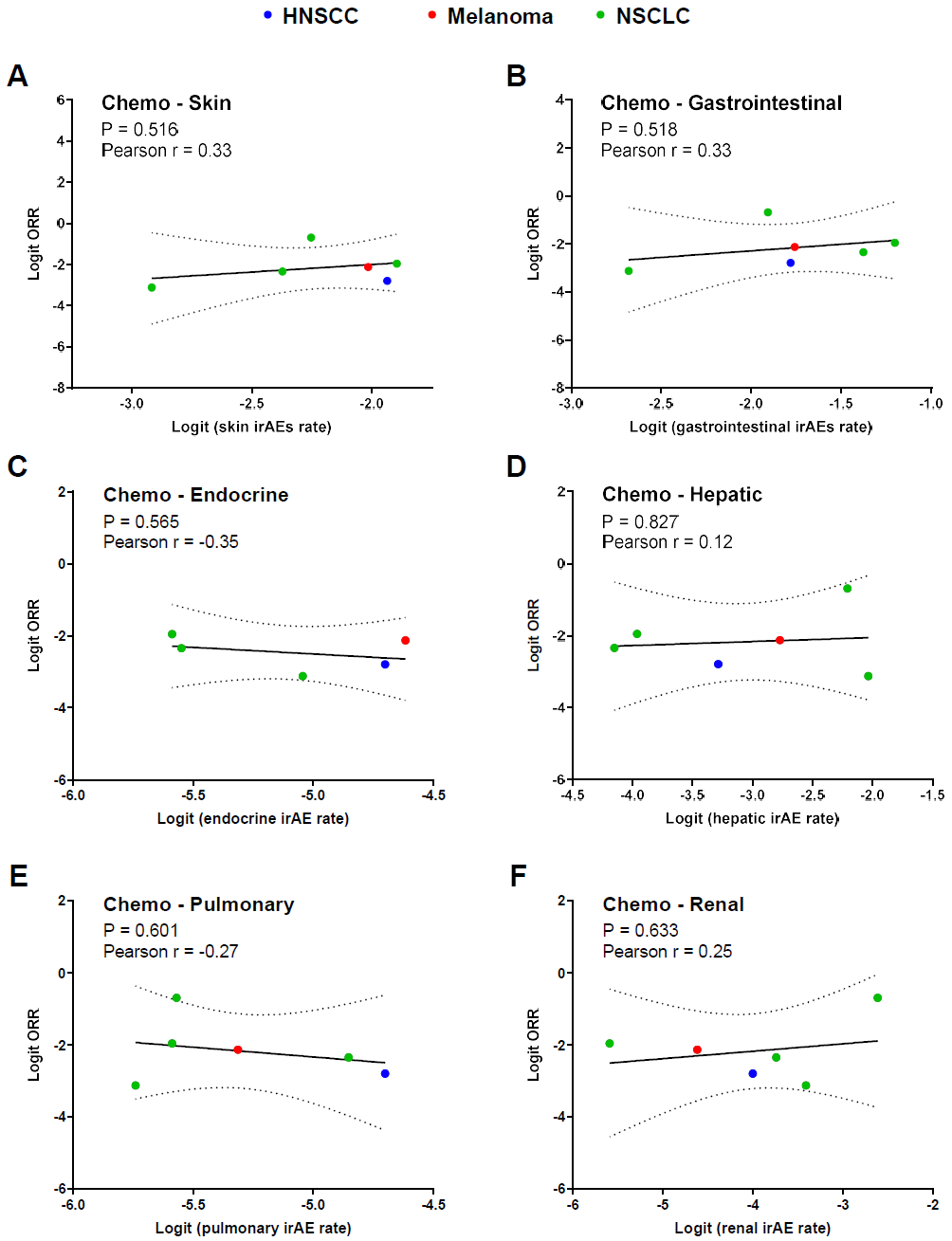


**Supplemental References:**

1. Topalian SL, Sznol M, McDermott DF, et al. Survival, durable tumor remission, and long-term safety in patients with advanced melanoma receiving nivolumab. *J Clin Oncol.* 2014;32(10):1020-1030.

2. Borghaei H, Paz-Ares L, Horn L, et al. Nivolumab versus Docetaxel in Advanced Nonsquamous Non-Small-Cell Lung Cancer. *N Engl J Med.* 2015;373(17):1627-1639.

3. Brahmer J, Reckamp KL, Baas P, et al. Nivolumab versus Docetaxel in Advanced Squamous-Cell Non-Small-Cell Lung Cancer. *N Engl J Med.* 2015;373(2):123-135.

4. McDermott DF, Drake CG, Sznol M, et al. Survival, Durable Response, and Long-Term Safety in Patients With Previously Treated Advanced Renal Cell Carcinoma Receiving Nivolumab. *J Clin Oncol.* 2015;33(18):2013-2020.

5. Hamanishi J, Mandai M, Ikeda T, et al. Safety and Antitumor Activity of Anti-PD-1 Antibody, Nivolumab, in Patients With Platinum-Resistant Ovarian Cancer. *J Clin Oncol.* 2015;33(34):4015-4022.

6. Larkin J, Chiarion-Sileni V, Gonzalez R, et al. Combined Nivolumab and Ipilimumab or Monotherapy in Untreated Melanoma. *N Engl J Med.* 2015;373(1):23-34.

7. Postow MA, Chesney J, Pavlick AC, et al. Nivolumab and ipilimumab versus ipilimumab in untreated melanoma. *N Engl J Med.* 2015;372(21):2006-2017.

8. Rizvi NA, Mazieres J, Planchard D, et al. Activity and safety of nivolumab, an anti-PD-1 immune checkpoint inhibitor, for patients with advanced, refractory squamous non-small-cell lung cancer (CheckMate 063): a phase 2, single-arm trial. *Lancet Oncol.* 2015;16(3):257-265.

9. Motzer RJ, Escudier B, McDermott DF, et al. Nivolumab versus Everolimus in Advanced Renal-Cell Carcinoma. *N Engl J Med.* 2015;373(19):1803-1813.

10. Motzer RJ, Rini BI, McDermott DF, et al. Nivolumab for Metastatic Renal Cell Carcinoma: Results of a Randomized Phase II Trial. *J Clin Oncol.* 2015;33(13):1430-1437.

11. Robert C, Long GV, Brady B, et al. Nivolumab in previously untreated melanoma without BRAF mutation. *N Engl J Med.* 2015;372(4):320-330.

12. Gettinger SN, Horn L, Gandhi L, et al. Overall Survival and Long-Term Safety of Nivolumab (Anti-Programmed Death 1 Antibody, BMS-936558, ONO-4538) in Patients With Previously Treated Advanced Non-Small-Cell Lung Cancer. *J Clin Oncol.* 2015;33(18):2004-2012.

13. Weber JS, D'Angelo SP, Minor D, et al. Nivolumab versus chemotherapy in patients with advanced melanoma who progressed after anti-CTLA-4 treatment (CheckMate 037): a randomised, controlled, open-label, phase 3 trial. *Lancet Oncol.* 2015;16(4):375-384.

14. Weber JS, Gibney G, Sullivan RJ, et al. Sequential administration of nivolumab and ipilimumab with a planned switch in patients with advanced melanoma (CheckMate 064): an open-label, randomised, phase 2 trial. *Lancet Oncol.* 2016;17(7):943-955.

15. Weber J, Gibney G, Kudchadkar R, et al. Phase I/II Study of Metastatic Melanoma Patients Treated with Nivolumab Who Had Progressed after Ipilimumab. *Cancer Immunol Res.* 2016;4(4):345-353.

16. Nishio M, Hida T, Atagi S, et al. Multicentre phase II study of nivolumab in Japanese patients with advanced or recurrent non-squamous non-small cell lung cancer. *ESMO Open.* 2016;1(4):e000108.

17. Sharma P, Callahan MK, Bono P, et al. Nivolumab monotherapy in recurrent metastatic urothelial carcinoma (CheckMate 032): a multicentre, open-label, two-stage, multi-arm, phase 1/2 trial. *Lancet Oncol.* 2016;17(11):1590-1598.

18. Gettinger S, Rizvi NA, Chow LQ, et al. Nivolumab Monotherapy for First-Line Treatment of Advanced Non-Small-Cell Lung Cancer. *J Clin Oncol.* 2016;34(25):2980-2987.

19. Antonia SJ, Lopez-Martin JA, Bendell J, et al. Nivolumab alone and nivolumab plus ipilimumab in recurrent small-cell lung cancer (CheckMate 032): a multicentre, open-label, phase 1/2 trial. *Lancet Oncol.* 2016;17(7):883-895.

20. El-Khoueiry AB, Sangro B, Yau T, et al. Nivolumab in patients with advanced hepatocellular carcinoma (CheckMate 040): an open-label, non-comparative, phase 1/2 dose escalation and expansion trial. *Lancet.* 2017;389(10088):2492-2502.

21. Carbone DP, Reck M, Paz-Ares L, et al. First-Line Nivolumab in Stage IV or Recurrent Non-Small-Cell Lung Cancer. *N Engl J Med.* 2017;376(25):2415-2426.

22. Hammers HJ, Plimack ER, Infante JR, et al. Safety and Efficacy of Nivolumab in Combination With Ipilimumab in Metastatic Renal Cell Carcinoma: The CheckMate 016 Study. *J Clin Oncol.* 2017;35(34):3851-3858.

23. Hellmann MD, Rizvi NA, Goldman JW, et al. Nivolumab plus ipilimumab as first-line treatment for advanced non-small-cell lung cancer (CheckMate 012): results of an open-label, phase 1, multicohort study. *Lancet Oncol.* 2017;18(1):31-41.

24. Overman MJ, McDermott R, Leach JL, et al. Nivolumab in patients with metastatic DNA mismatch repair-deficient or microsatellite instability-high colorectal cancer (CheckMate 142): an open-label, multicentre, phase 2 study. *Lancet Oncol.* 2017;18(9):1182-1191.

25. Yamazaki N, Kiyohara Y, Uhara H, et al. Efficacy and safety of nivolumab in Japanese patients with previously untreated advanced melanoma: A phase II study. *Cancer Sci.* 2017;108(6):1223-1230.

26. Sharma P, Retz M, Siefker-Radtke A, et al. Nivolumab in metastatic urothelial carcinoma after platinum therapy (CheckMate 275): a multicentre, single-arm, phase 2 trial. *Lancet Oncol.* 2017;18(3):312-322.

27. Kudo T, Hamamoto Y, Kato K, et al. Nivolumab treatment for oesophageal squamous-cell carcinoma: an open-label, multicentre, phase 2 trial. *Lancet Oncol.* 2017;18(5):631-639.

28. Hida T, Nishio M, Nogami N, et al. Efficacy and safety of nivolumab in Japanese patients with advanced or recurrent squamous non-small cell lung cancer. *Cancer Sci.* 2017;108(5):1000-1006.

29. Morris VK, Salem ME, Nimeiri H, et al. Nivolumab for previously treated unresectable metastatic anal cancer (NCI9673): a multicentre, single-arm, phase 2 study. *Lancet Oncol.* 2017;18(4):446-453.

30. Kang YK, Boku N, Satoh T, et al. Nivolumab in patients with advanced gastric or gastro-oesophageal junction cancer refractory to, or intolerant of, at least two previous chemotherapy regimens (ONO-4538-12, ATTRACTION-2): a randomised, double-blind, placebo-controlled, phase 3 trial. *Lancet.* 2017;390(10111):2461-2471.

31. Omuro A, Vlahovic G, Lim M, et al. Nivolumab with or without ipilimumab in patients with recurrent glioblastoma: results from exploratory phase I cohorts of CheckMate 143. *Neuro Oncol.* 2018;20(5):674-686.

32. Ma BBY, Lim WT, Goh BC, et al. Antitumor Activity of Nivolumab in Recurrent and Metastatic Nasopharyngeal Carcinoma: An International, Multicenter Study of the Mayo Clinic Phase 2 Consortium (NCI-9742). *J Clin Oncol.* 2018;36(14):1412-1418.

33. Long GV, Atkinson V, Lo S, et al. Combination nivolumab and ipilimumab or nivolumab alone in melanoma brain metastases: a multicentre randomised phase 2 study. *Lancet Oncol.* 2018;19(5):672-681.

34. Tawbi HA, Forsyth PA, Algazi A, et al. Combined Nivolumab and Ipilimumab in Melanoma Metastatic to the Brain. *N Engl J Med.* 2018;379(8):722-730.

35. Wolchok JD, Kluger H, Callahan MK, et al. Nivolumab plus ipilimumab in advanced melanoma. *N Engl J Med.* 2013;369(2):122-133.

36. Lee JS, Lee KH, Cho EK, et al. Nivolumab in advanced non-small-cell lung cancer patients who failed prior platinum-based chemotherapy. *Lung Cancer.* 2018;122:234-242.

37. Quispel-Janssen J, van der Noort V, de Vries JF, et al. Programmed Death 1 Blockade With Nivolumab in Patients With Recurrent Malignant Pleural Mesothelioma. *J Thorac Oncol.* 2018;13(10):1569-1576.

38. Caushi JX, Chan HY, Sidhom JW, et al. Nivolumab plus Ipilimumab in Lung Cancer with a High Tumor Mutational Burden. *N Engl J Med.* 2018;378(22):2093-2104.

39. Overman MJ, Lonardi S, Wong KYM, et al. Durable Clinical Benefit With Nivolumab Plus Ipilimumab in DNA Mismatch Repair-Deficient/Microsatellite Instability-High Metastatic Colorectal Cancer. *J Clin Oncol.* 2018;36(8):773-779.

40. Motzer RJ, Tannir NM, McDermott DF, et al. Nivolumab plus Ipilimumab versus Sunitinib in Advanced Renal-Cell Carcinoma. *N Engl J Med.* 2018;378(14):1277-1290.

41. D'Angelo SP, Mahoney MR, Van Tine BA, et al. Nivolumab with or without ipilimumab treatment for metastatic sarcoma (Alliance A091401): two open-label, non-comparative, randomised, phase 2 trials. *Lancet Oncol.* 2018;19(3):416-426.

42. Janjigian YY, Bendell J, Calvo E, et al. CheckMate-032 Study: Efficacy and Safety of Nivolumab and Nivolumab Plus Ipilimumab in Patients With Metastatic Esophagogastric Cancer. *J Clin Oncol.* 2018;36(28):2836-2844.

43. Wu YL, Lu S, Cheng Y, et al. Nivolumab Versus Docetaxel in a Predominantly Chinese Patient Population With Previously Treated Advanced NSCLC: CheckMate 078 Randomized Phase III Clinical Trial. *J Thorac Oncol.* 2019;14(5):867-875.

44. Lebbe C, Meyer N, Mortier L, et al. Evaluation of Two Dosing Regimens for Nivolumab in Combination With Ipilimumab in Patients With Advanced Melanoma: Results From the Phase IIIb/IV CheckMate 511 Trial. *J Clin Oncol.* 2019;37(11):867-875.

45. Disselhorst MJ, Quispel-Janssen J, Lalezari F, et al. Ipilimumab and nivolumab in the treatment of recurrent malignant pleural mesothelioma (INITIATE): results of a prospective, single-arm, phase 2 trial. *Lancet Respir Med.* 2019;7(3):260-270.

46. Ready N, Hellmann MD, Awad MM, et al. First-Line Nivolumab Plus Ipilimumab in Advanced Non-Small-Cell Lung Cancer (CheckMate 568): Outcomes by Programmed Death Ligand 1 and Tumor Mutational Burden as Biomarkers. *J Clin Oncol.* 2019;37(12):992-1000.

47. Ferris RL, Blumenschein G, Jr., Fayette J, et al. Nivolumab for Recurrent Squamous-Cell Carcinoma of the Head and Neck. *N Engl J Med.* 2016;375(19):1856-1867.

48. Scherpereel A, Mazieres J, Greillier L, et al. Nivolumab or nivolumab plus ipilimumab in patients with relapsed malignant pleural mesothelioma (IFCT-1501 MAPS2): a multicentre, open-label, randomised, non-comparative, phase 2 trial. *Lancet Oncol.* 2019;20(2):239-253.
